# Prevalence and correlates of medical student mistreatment in Nigeria: A narrative review

**DOI:** 10.1097/MD.0000000000037747

**Published:** 2024-04-12

**Authors:** Emmanuel Aniekan Essien, Bonaventure Michael Ukoaka, Faithful Miebaka Daniel, Gideon Okobru, Tajuddeen Wali Adam

**Affiliations:** a Research and Training Unit, Federal Neuropsychiatric Hospital, Calabar, Nigeria; b Community and Clinical Research Division, First On-Call Initiative, Port Harcourt, Nigeria; c Community and Clinical Research Division, First On-Call Initiative, Kharkiv, Ukraine; d University of Calabar, Calabar, Nigeria; e College of Health Science, Bayero University Kano, Kano, Nigeria.

**Keywords:** medical education, medical students, mental health, Nigeria, physical abuse

## Abstract

Mistreatment in medical education encompasses various forms of abusive behavior, often indicating a disregard for students’ dignity and interfering with the learning process. This review paper aims to investigate the prevalence, patterns, and correlates of medical student mistreatment in Nigerian medical education and shed light on its impact on students’ well-being. A literature search was conducted in August 2023 using Medline, Google Scholar, and Web of Science databases to identify relevant studies on the mistreatment of Nigerian medical students. Inclusion criteria encompassed all studies written in English, regardless of study design, while editorials, reviews, and opinion articles were excluded. Six studies with a total sample size of 1432 were included in the review. The review revealed high mistreatment rates, ranging from 46% to 91%, with verbal abuse being the most common form. Male students were more likely to experience mistreatment, while females had higher rates of sexual abuse. Perpetrators included male and female healthcare professionals, with consultants and resident doctors as common perpetrators. Age and study level were associated with mistreatment experiences. Consequences of mistreatment included emotional distress, depression, loss of self-confidence, academic disillusionment, substance abuse, and suicidal thoughts. Reporting rates were low due to a lack of awareness, fear of reprisal, and perceived futility. The findings underscore the need for institutions to create a supportive environment, raise awareness of available support systems, and implement policies to prevent mistreatment. Future research should focus on larger-scale studies with diverse samples and longitudinal designs to address limitations in the current literature.

## 1. Introduction

Medicine is among the most challenging academic programs in various climes. Not only is admission more competitive, but the associated intellectual, psychological, physical, and financial demands can negatively impact the quality of life during the program.^[1]^ Medical education encompasses all aspects of formal training for medical undergraduates and postgraduate specialist training. The structure varies in different countries, but Nigeria medical education is similar in design to British medical education, which influenced the establishment of the first center for training at University College Hospital, Ibadan.^[2]^

Mistreatment in medical education refers to any form of abusive behavior by an educator, often indicating a disregard for students’ dignity and interfering with the learning process.^[3]^ Medical student mistreatment includes abuse in any form experienced by medical students from anyone within the academic environment.^[3]^ The current understanding of medical student mistreatment was first brought to light by Henry Silver, who compared it to child abuse in his 1982 article. He stated that after starting their studies, medical students “become cynical, some dejected, others frightened or depressed, and a few filled with frustration.”^[4]^ Cases reported in Nigeria range from physical abuse to public belittlement and humiliation and often come in the form of verbal or physical abuse, discrimination, perception of being excluded, exploitation, and sexual abuse, among others.^[3]^

Much like the military, medical training and practice are hierarchical, and those in upper echelons wield significant influence over the pedagogical approach, sometimes with arbitrary discretionary powers.^[5]^ This hierarchical structure is considered causal in mistreatment and academic witch-hunts, affecting both undergraduate and postgraduate medical education.^[1,5,6]^ In the United States, mistreatment is regularly surveyed at graduation and found to be high despite concerns about underreporting.^[6,7]^ Considerable resources have been invested, especially in developed countries, into curtailing this phenomenon; however, the prevalence remains high worldwide.^[5]^ Its perpetuation may be attributed to mistreated students adopting similar teaching methods when they become doctors, establishing a “cycle of abuse.”^[5]^

Many medical educators lack formal instruction on effective educational techniques, which might explain their defaulting to the teaching methods they experienced.^[8]^ The Socratic Method, an age-old practice of learning that involves asking questions to assess a student knowledge before imparting answers and stimulating further learning, is a prime example.^[8]^ While some instructors contend that this approach is beneficial, requiring students to prepare in advance, others argue that it may be flawed.^[8]^ The lack of evidence-based guidance for the teaching process creates room for individual idiosyncrasies, inadvertently fostering mistreatment.^[8]^

This persisting phenomenon harms learning, student well-being and career decisions.^[6]^ Reports indicate harmful repercussions on students’ mental, social, and psychological well-being.^[9]^ It negatively impacts students’ academic performance, social interactions with peers, personality, and preferences for post-graduate specializations.^[6,10]^ Furthermore, it has been associated with enduring mental health consequences, including binge drinking, depression, and suicide attempts.^[10]^ Besides academic burnout, medical mistreatment is linked to medical school dropouts, regrets about choosing a medical career, and poor career satisfaction.^[6]^ Medical student mistreatment has been insufficiently explored in Nigeria, yet it continues to undermine the quality of medical education in the country.^[1]^

This paper aims to shed light on the burden of this pervasive phenomenon in Nigeria and thus raise awareness of the need to curb it to foster a conducive medical learning environment. Our objective was to identify and summarize the prevalence, pattern, and correlates of medical student mistreatment in the Nigerian medical education sector.

## 2. Methods

### 2.1. Search strategy

A literature search using online databases was conducted to identify relevant Nigerian studies. The search on Medline was done using the MESH terms “students, medical,” “harassment, non-sexual” “dehumanization,” “scapegoating,” “social discrimination,” “social marginalization,” “emotional abuse,” “shame,” “bullying,” and “Nigeria.” Google Scholar and the Web of Science databases were also searched for relevant articles. The search was without restriction regarding publication date and was conducted in August 2023.

### 2.2. Eligibility criteria

Inclusion criteria: All prior original studies (regardless of study design) on the mistreatment of Nigerian medical students written in English. Editorials, reviews, and opinion articles were excluded.

### 2.3. Data extraction

The extracted data from each study included the prevalence and pattern of mistreatment, the associated factors, perceptions and responses to mistreatment. The author names, publication dates, sample size and study design were also extracted (Table 1).

**Table 1:** Summary of reviewed papers.

SN	First author	Yr	Study design and location	Sample size/participation by gender	Summary of results
1	Olasoji et al^[8]^	2018	Mixed Method study design/University of Maiduguri, Borno State, Nigeria.	47/25 male and 22 female.	Prevalence: 50% occasionally, 40% once, 10% frequently Opinions: 60% believed it causes adverse effects, 43.8% considered it part of education, 37.5% said it makes 1 stronger, 0% thought they would do the same if they were teachers Reasons for Not Reporting: Didn’t seem necessary (43.8%), Nothing would be done (25%), Feared reprisal (18.8%), Didn’t know what to do (18.8%)
2	Owoaje et al^[11]^	2012	Cross-Sectional Study/University of Ibadan, Oyo state, Nigeria.	269/165 male and 104 females.	Common Mistreatment: Verbal (92.6%), Humiliation (87.4%), Disparaging remarks (71.4%) Sexual Harassment characteristics: Denied opportunities (16.7%), Favoritism (16.4%), Poor evaluations (12.6%), Sexual advances (10%) Effects: Caused stress (64.0%), Affected relationships (63.4%), Reduced self-confidence (45.4%), Caused depression (40.5%), Affected academic performance (24.4%), Sleep problems (22.1%), Career choice regrets (18.7%), Resorted to alcohol (7.7%), Resorted to smoking (5.7%), Considered suicide (1.9%)
3	Oji et al^[12]^	2000	Cross-Sectional Study/University of Nigeria, Enugu State, Nigeria	231 responses/Nil gender data	Prevalence/Forms of Abuse: Verbal abuse (74%), Psychological abuse (55.8%), Intimidation (50.2%), Unnecessary medical risk (47.1%), Physical abuse (6%), Ethnic abuse (5.2%), Religious Abuse (5.9%), Sexual abuse (0.9%)
4	Sesugh et al^[13]^	2022	Cross-Sectional Study/Six geopolitical zones in Nigeria.	319/ 141 males and 178 Females	Gender-Based Abuse: Verbal abuse (46.5%), Sexual abuse (34.2%), Physical abuse (19.4%) Perpetrators: Consultants/Lecturers (58.3%), Registrars (17.2%) Gender of perpetrators: Male (54.6), Female (20.7%), Both (24.7%)
5	Oku et al^[1]^	2014	Cross-Sectional Study/Cross River State, Nigeria.	451/288 males and 163 females.	Forms of Mistreatment: verbal abuse (52.5%), Inappropriate behavior (25%), Being excluded or ignored (9.4%), Physical abuse (7.5%), Sexual abuse (3.8%), Written abuse (1.8%) Most Frequent Perpetrators: Verbal abuse (Consultants 18.6%), Physical Abuse (50%), Inappropriate Behavior (Lecturers 34.6%), Written abuse (Students 62.1%), Excluded or ignored (Students 23.7%), Sexual Abuse (Lecturers/Nurses 30.8%)
6	Adayonfo et al^[14]^	2019	Cross-Sectional Study/Benin City, Edo State, Nigeria.	72 analyzed/ 47 males and 25 females.	Prevalence of Mistreatment 30 days vs past year: Verbal (19.4%) vs (26.4%), Physical (16.7%) vs (18.1%) Perpetrators of Mistreatment 30 days vs past year: Lecturers/Trainers (25%) vs (38.5%), Neighbours (8.3) vs (7.7%)

#### 2.3.1. Included studies

Six studies were included in the final narrative synthesis.

### 2.4. Study outcome

Outcomes of interest were the prevalence, characterization, sources, and factors associated with medical student mistreatment in Nigeria.

#### 2.4.1. Ethical approval

Since this was a literature review, ethical approval was not required.

## 3. Findings

### 3.1. Characteristics of included studies

Six cross-sectional (5 quantitative and 1 mixed methods) studies with a total sample size of 1432 conducted from 2012 to 2022 were included. Data were collected through self-administered questionnaires, except for the mixed-methods study, which also conducted interviews.

### 3.2. Prevalence and pattern of mistreatment

Mistreatment rates were high, ranging from 46% to 91%. Five studies examined the types of mistreatment, and verbal abuse was the most common.^[1,11–14]^ Physical abuse was the least type of abuse in 2 studies,^[13,14]^ while sexual abuse,^[12]^ written abuse,^[1]^ and being asked to do something immoral or unethical,^[11]^ were the least in 3 studies. Four studies assessed sexual abuse, and the highest prevalence was 34.2%,^[13]^ with the other studies reporting rates of 3.8%,^[1]^ 10^11^ and 0.9%.^[12]^ Except for sexual harassment, male students were more likely to experience abuse.^[1,11,12]^

### 3.3. Perpetrators

While female nurses were found to mistreat female medical students more frequently, male students often experienced abuse from healthcare professionals of both genders.^[1,11]^ According to Sesugh, the number of male perpetrators (54.6%) was significantly higher than females (20.7%).^[13]^ Lecturers/consultants and resident doctors were the commonest perpetrators.^[1,13]^ In addition, Owoaje noted that sexual abuse was perpetrated the most by residents (27.5%) and consultants (25.2%).^[11]^ Adayanfo et al reported a lower proportion of lecturers and seniors as perpetrators than Oku et al and Sesugh.^[14]^

### 3.4. Factors influencing mistreatment

#### 3.4.1. Age

In the reviewed studies, limited attention was given to the relationship between age and the experience of mistreatment, except for the study by Oku et al.^[1]^ Age was a contributing factor to the experience of mistreatment, with a significantly higher proportion of individuals aged 25 and above (52.3%) reporting mistreatment compared to those below 25 years (28.8%).^[1]^

#### 3.4.2. Level of study

Only Oku et al^[1]^ explored the relationship between the study level and mistreatment. The proportion increased as students progressed to clinical levels, from 11% among first-years to 53.6% among fifth-year students; the finding was also statistically significant.

#### 3.4.3. Gender

Owoaje^[11]^ found that male respondents reported slightly higher abusive incidents across at least 4 categories. However, this difference was not significant.^[11]^ The survey revealed that male students (41.2%) were more likely to receive threats of failure or low marks compared to their female counterparts (23.1%). On the other hand, female students (40.4%) reported a higher incidence of sexual harassment compared to males (29.7%). They were also more likely to experience unwanted sexual advances.^[11]^

#### 3.4.4. Effects

Previous research indicates mistreatment causes distress to many students, particularly their physical, emotional, and social well-being. For instance, Oji et al^[12]^ reported emotional distress in over half of mistreated students. Olasoji^[8]^ discovered that most respondents reported having experienced mistreatment that caused significant stress and negatively affected their relationship with the perpetrators. These results align with the findings by Owoaje et al^[11]^ where almost two-thirds of participants reported a form of mistreatment. Also, Olasoji^[8]^ reported that 60% of respondents suffered adverse effects due to mistreatment.

Over 20% of respondents in the study by Oji et al^[12]^ stated that they would not have chosen medicine had they known about the extent of mistreatment; only 6% reported that they would abandon the profession after qualification due to the related stress.

In a study conducted by Owoaje et al,^[11]^ it was found that over 40% of respondents experienced depression and a loss of self-confidence due to mistreatment, which caused them to feel worse off than their peers in other professions. Additionally, almost a quarter of participants became more cynical about academics and the medical profession. Based on the research conducted by Adayonfo et al,^[14]^ it was found that individuals who experienced mistreatment were more likely to consume and abuse alcohol. Buttressing the consequences, Owoaje et al^[11]^ reported that 18.7% of participants regretted their choice of medicine and resorted to unhealthy coping mechanisms, such as alcohol (7.7%) and smoking (5.7%). Mistreatment was also linked to suicidal thoughts in 1.9% of male participants, which is a unique finding not reported in other studies.^[11]^

### 3.5. Responses to mistreatment

Oku et al^[1]^ found that only 8.8% of mistreatment victims reported their experiences, with nearly 50% choosing to do nothing about incidents. Among those who spoke up, 40.6% discussed the matter with family and friends, with colleagues and close friends being the most common confidants. About 90% of participants were unaware of any support for mistreatment victims. Similarly, Sesugh^[13]^ found that 91% of those who experienced gender discrimination did not report it. Olasoji^[8]^ discovered that 60% of respondents disclosed their experiences to someone, slightly higher than Oku et al’s findings. Olasoji^[8]^ also found that 43.8% of non-reporters felt it was not important enough to report, 25% believed nothing would be done about it, and 18.8% did not know what to do or feared reprisal. Furthermore, Olasoji^[8]^ found that 66.7% of respondents were neutral about their satisfaction after disclosing their experiences, with over half feeling more dissatisfied than satisfied.

## 4. Discussion

The review aimed to report on the prevalence and correlates of mistreatment experienced by medical students in Nigeria. The paper focused on previous research conducted in Nigeria to explore the realities of medical students undergoing medical education across various Nigerian Medical Schools. We found that mistreatment rates ranged from 46% to 91%. Verbal abuse was the most common form, and male students were more likely to experience mistreatment; however, females had higher rates of sexual abuse. Perpetrators were male and female healthcare professionals, lecturers, consultants, and resident doctors. Age and study levels were associated with mistreatment experiences. Several consequences of mistreatment were reported, including emotional distress, stress, depression, loss of self-confidence, academic disillusionment, substance abuse, and worryingly suicidal thoughts. However, reporting rates remained low, with a lack of awareness, fear of reprisal, and perceived futility hindering students from seeking help.

The reviewed literature showed that the prevalence of mistreatment was high among medical students in Nigeria, and this is consistent with other countries like the United States (75%), Pakistan (66.2%), and Thailand (63.4%).^[12]^ This underscores the large-scale nature of medical mistreatment, which is common across different countries due to various factors. The reviewed studies found that verbal abuse and feelings of exclusion were more frequently reported by about 74% of respondents in Nigeria, which is consistent with findings from other countries.^[12]^ Sexual harassment rates varied widely across various studies, ranging between 0.9% and 34.2% in selected studies.^[12,13]^

A consistent trend in the reviewed papers was that consultants practised more forms of mistreatment, either verbal abuse (shouting at subjects, public humiliation, or belittlement) or physical and sexual abuse, than the resident doctors or any other cadre of health professionals. Although our findings are consistent with global studies, some contrasting reports exist.^[15,16]^ They reported that more respondents suffered mistreatment from residents than faculty members, while Cook et al reported 68.9% mistreatment from both categories of perpetrators.^[6]^ Residents tend to replicate the same teaching methodology used by their trainers (the Consultants) during ward rounds at their after-school clinical contacts with medical students. Residents, who are themselves still undergoing medical education, might be replicating the teaching styles of their supervising consultants, explaining their high mistreatment of medical students.

Only 2 of the 6 studies reviewed reported a gender-based risk factor; however, previous studies also link gender to mistreatment risk.^[15]^ One study noted a reversal of pattern in their report where more female students suffered more verbal and sexual abuse and reported more incidents as well.^[17]^ The pattern of gendered associations we found might be related to gender norms in the local context. The level of study was associated with mistreatment in only one reviewed study; however, this is consistent with previous research.^[1,18]^ This could be because the other papers mainly focused on the higher classes that have more clinical teaching encounters and, therefore, more opportunities for mistreatment to occur.

Other research also notes the severe consequences of mistreatment on the well-being, academic performance, and career choices of students, leading to stress, anxiety, and depression, which affects their mental health.^[6,16]^ Mistreated students may struggle to focus on their studies, resulting in poor academic performance. It also impacts their career choices, with some students becoming disenchanted and considering dropping out of medical school.^[6,16]^ Mistreatment discourages students from pursuing careers in academic medicine, which affects medical research and education.^[6,16]^ The effects of mistreatment can also be long-lasting, resembling post-traumatic stress disorder.^[6,16]^

## 5. Conclusion and recommendations

Mistreatment of medical students is a serious issue that often goes unreported due to various reasons like fear of negative consequences and a lack of confidence in the available support systems. It creates a culture of mistreatment and deprives the victims of the required support. There is also a lack of awareness about the available support systems. To address this, institutions must make these systems more visible, create a respectful and empathetic culture, and train faculty and staff to recognize and address mistreatment. This would encourage more students to come forward and seek help, leading to effective interventions and a better learning environment for everyone involved.^[6]^

Recent studies have shown that medical students who experience mistreatment often do not report it.^[1,8]^ Those who do, often confide in close friends, colleagues, or family members, indicating a lack of confidence in the official support systems. In addition, many medical students are not aware of the support systems in place to address mistreatment, which discourages them from reporting incidents and seeking help. Although some students may view “toxic” practices as usual, and helpful in learning or strengthening their resilience, it is still essential to create a supportive environment for those who encounter mistreatment. Policies should be introduced to prevent any form of abuse, mistreatment, or public humiliation of medical students and trainees, which aligns with global efforts.^[5,19]^ Universities should include protection against all forms of academic victimization in their laws. This would encourage the reporting of all mistreatment of medical students, as this is consistently underreported in local and international studies.

Previous studies have highlighted the impact of mistreatment on medical students, but they are limited in their scope due to small sample sizes, geographic constraints, and retrospective cross-sectional designs.^[1,6]^ Future research should aim for larger-scale studies with more diverse samples and longitudinal strategies to address these limitations. Additionally, future research should consider multiple perspectives, such as faculty and peers, to gain a more comprehensive understanding of mistreatment experiences. Further exploration of the underlying causes of mistreatment in medical education is necessary to develop targeted interventions. To truly understand the long-term impact of injustice, it is crucial to conduct more longitudinal studies that follow medical students throughout their education and into their careers. Ultimately, addressing the limitations of current research and emphasizing future research initiatives can assist in effectively managing and combating the mistreatment of medical students. Creating a supportive and respectful environment is critical for the well-being and success of future healthcare professionals, which has significant consequences for the overall quality of patient care.

Managing the self-perpetuating cycle of medical mistreatment is of crucial interest because of the far-reaching implications on academics, general well-being, self-confidence, psychosocial coping, and career aspirations. The high prevalence rates should stimulate conversations and galvanize interest to address the challenge, especially for low-resource settings, hoping to inspire confidence in a motivated workforce. Also, the patterns of abuse and their perpetrators offer us insights into possible targets in our intervention. A critical evaluation of factors underlying the experience of mistreatment enables the understanding of differential experiences that may suggest subtle systemic prejudice. The perception of the effects of abuse and the responses of victims provide clues to help victims, avert long-term implications, and increase accountability among perpetrators. The low reporting rates and lack of awareness about support systems underscore the need to create a more supportive environment that would mitigate the culture of silence. Addressing the limitations of existing research and focusing on future directions will enable us to gain a deeper understanding of this critical issue and implement effective measures to combat mistreatment in medical education.

## Author contributions

**Conceptualization:** Emmanuel Aniekan Essien, Bonaventure Michael Ukoaka, Faithful Miebaka Daniel.

**Data curation:** Emmanuel Aniekan Essien, Bonaventure Michael Ukoaka, Faithful Miebaka Daniel.

**Methodology:** Emmanuel Aniekan Essien, Bonaventure Michael Ukoaka, Faithful Miebaka Daniel.

**Project administration:** Emmanuel Aniekan Essien.

**Resources:** Emmanuel Aniekan Essien, Bonaventure Michael Ukoaka, Faithful Miebaka Daniel.

**Supervision:** Emmanuel Aniekan Essien.

**Validation:** Emmanuel Aniekan Essien, Bonaventure Michael Ukoaka, Faithful Miebaka Daniel.

**Visualization:** Emmanuel Aniekan Essien.

**Writing – original draft:** Emmanuel Aniekan Essien, Bonaventure Michael Ukoaka, Faithful Miebaka Daniel, Gideon Okobru, Tajuddeen Wali Adam.

**Writing – review & editing:** Emmanuel Aniekan Essien, Bonaventure Michael Ukoaka, Faithful Miebaka Daniel, Gideon Okobru, Tajuddeen Wali Adam.
